# From Classical Enamel/Dentin Bonding to Self-Adhesive Composites: A Narrative Review of Current Clinical Aspects

**DOI:** 10.3390/bioengineering13070760

**Published:** 2026-06-29

**Authors:** Andreas Rathke, Sigmar Schnutenhaus, Rainer Seemann

**Affiliations:** 1 Faculty of Medicine, University of Ulm, 89081 Ulm, Germany; 2 Clinical Research, Dentsply Sirona, 78464 Konstanz, Germany; 3 Department of Prosthetic Dentistry, Center of Dentistry, University of Ulm, 89081 Ulm, Germany; 4 Center for Dentistry MVZ GmbH, 78247 Hilzingen, Germany; 5 Department of Restorative, Preventive and Pediatric Dentistry, zmk Bern, University Bern, 3010 Bern, Switzerland

**Keywords:** adhesive, bonding, clinical performance, non-systematic review, self-adhesive composite, simplification

## Abstract

Adhesive dentistry has undergone a significant evolution, transitioning from classical multi-step adhesives to simplified single-bottle or universal adhesives and now towards self-adhesive composites that no longer require a separate adhesive. This review focused on clinical applications, recent developments, and future trends in peer-reviewed articles identified through database searches in PubMed, Web of Science Core Collection, EBSCO DOSS, and Google Scholar without date restrictions. Reference mining of the identified articles was also used. Significant advances in the clinical performance of simplified adhesives are challenging the status of “gold standard” adhesives, although their long-term reliability in load-bearing (Class II) composite restorations remains uncertain due to a lack of longitudinal real-world data and extended survival metrics. Integration of acidic functional monomers like 10-MDP is becoming a standard to create a more stable chemical bond with dentin hydroxyapatite, rather than relying solely on micromechanical retention. Ensuring long-term stability of the hybrid layer against degradation remains a challenge. When enamel is present, etching with phosphoric acid is still recommended, while the necessity of enamel beveling is being questioned. As the clinical success of self-adhesive flowable composites is limited to non-load-bearing areas, further developments are moving toward self-adhesive bioactive composites for bulk-filling that combine self-adhesive properties with load-bearing capacity.

## 1. Introduction

**Figure 1:**
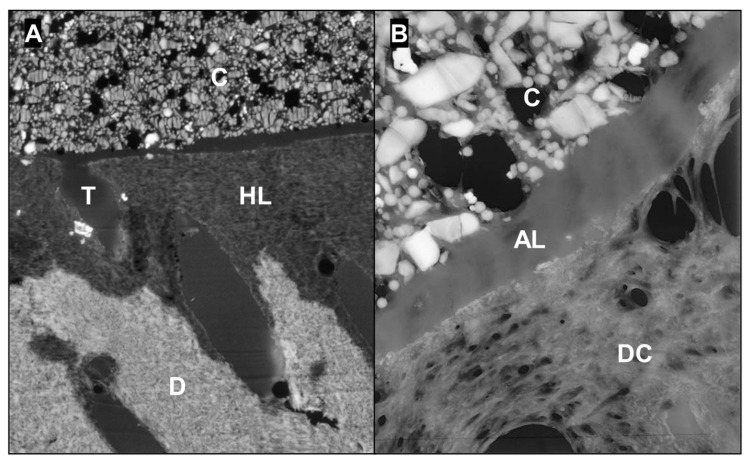
Transmission electron microscopy (TEM) photomicrographs of the adhesive-dentin interface following application of an etch-and-rinse (ER) adhesive: (**A**) Overview TEM interfacial ultrastructure, showing distinct resin tags along with a thick hybrid layer (×1250 magnification). (**B**) High magnification, detailing the interpenetrating resin network with collagen fibrils (×16,000 magnification). AL: Adhesive layer; D: Dentin; HL: Hybrid layer; C: Composite; DC: Dentin collagen; T: Resin tag (Courtesy of the Central Facility for Electron Microscopy, University of Ulm, Ulm, Germany).

Adhesive restorations made of resin-based composites or ceramics are widely used in dentistry because they have long-term survival, are tooth colored, and enable minimally invasive or defect-oriented preparations that rely less on macro-mechanical retention and resistance form. Due to current legislation, amalgam restorations are banned in several countries and are being phased out in others, making direct composite restorations a successful alternative to amalgam. The application of resin-based adhesives is essential for their clinical success, as it ensures adhesion between the composite and the tooth substrates. The mechanism of adhesion is based primarily on micro-mechanical retention to the acid-etched enamel and the formation of a hybrid layer with dentin (Figure 1).

**Figure 2:**
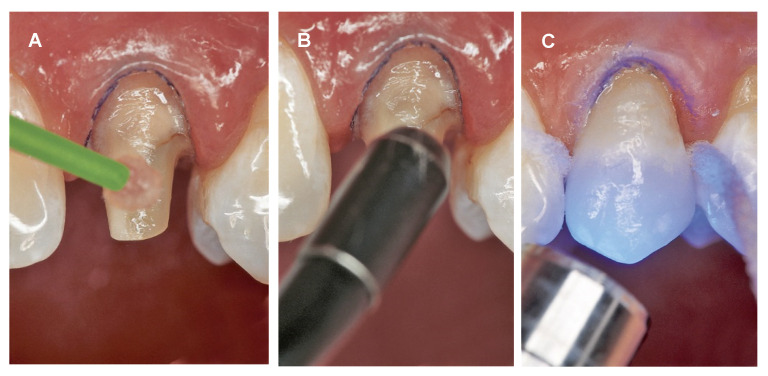
Bonding procedure when using the self-etch (SE) mode: (**A**) Application of the adhesive. (**B**) Evaporation of the solvent. (**C**) Light curing after placement of the glass–ceramic crown.

**Figure 3:**
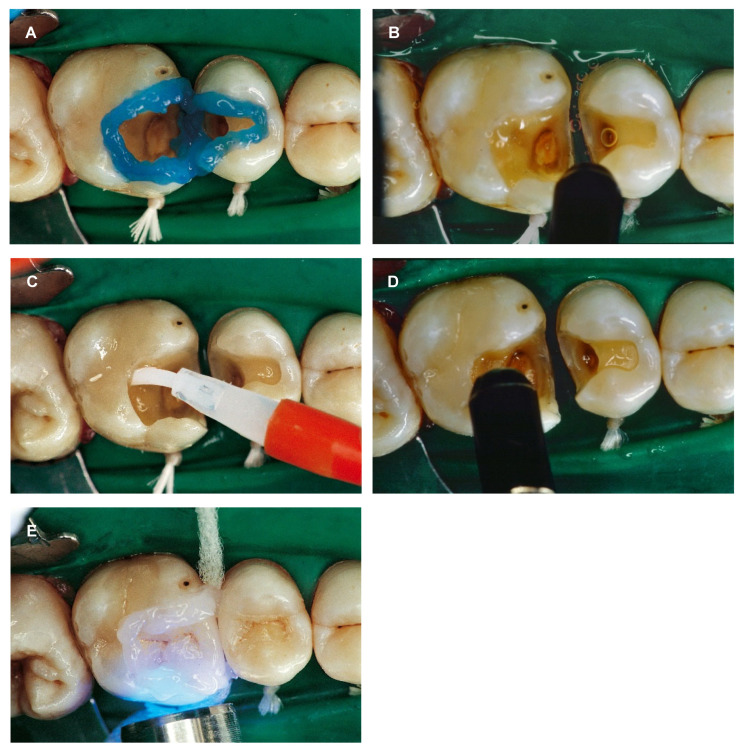
Bonding procedure when using the combined ER/SE mode: (**A**) Selective etching of enamel margins with phosphoric acid. (**B**) Rinsing with air-water spray. (**C**) Application of the adhesive in SE mode. (**D**) Evaporation of the solvent. (**E**) Light curing of the restoration.

Today’s adhesives follow an etch-and-rinse (ER) or self-etch (SE) bonding strategy. While ER adhesives require phosphoric acid to etch enamel and dentin, SE adhesives contain acidic functional monomers that in principle simultaneously etch (demineralize) and infiltrate the tooth substrates, thus eliminating the separate etching and rinsing step. The latest generation of universal or multimode adhesives has been developed for use in both ER and SE bonding modes but is also compatible with the combined mode, in which the enamel is selectively etched with phosphoric acid and the adhesive is applied to the etched enamel and dentin in SE mode (so-called selective enamel etching (SEE)). This allows clinicians to select the appropriate mode according to their personal preference and/or depending on the clinical situation. For example, the use of SE mode is considered advantageous for bonding to dentin and/or gingival margins (Figure 2), while additional etching with phosphoric acid is recommended when enamel margins are present (Figure 3).

Multiple universal adhesives are suitable not only for bonding to enamel and dentin but also as adhesive primers on indirect restorative materials such as ceramics, metal alloys, and composites. Although manufacturers typically state that universal adhesives can be used even with suboptimal moisture control, enamel and dentin bonding remain time-consuming and technique sensitive steps in the restorative procedure. Failures at the adhesive interface can lead to a cascade of undesirable effects such as postoperative hypersensitivity, marginal gaps, and marginal leakage [1,2,3]. The occurrence of secondary caries and fractures in bulk and margins has been identified in clinical studies as the main failures of composite restorations [1,2,3]. This therefore requires a comprehensive understanding of dental bonding; consequently, the present narrative review aims to highlight current clinical aspects in this field and explore future perspectives.

## 2. Materials and Methods

The non-systematic review followed the recommendations of the Scale for the Assessment of Narrative Review Articles (SANRA) [4]. The aim was to provide a descriptive overview of current developments in adhesive dentistry in the application of composite restorations. A targeted literature search was conducted across PubMed, Web of Science Core Collection, EBSCO Dentistry & Oral Sciences Source (DOSS), and Google Scholar without publication date limits (search period: March–April 2026). Relevant publications were identified using keywords such as “composite”, “bonding”, “etch-and-rinse”, “self-etch”, “universal adhesive”, and “self-adhesive”. Search parameters included title/abstract field tags (e.g., [tiab] in PubMed) and publication type filters such as reviews and clinical trials, with a restriction to peer-reviewed articles and the English language. This targeted strategy yielded 245 initial search records in PubMed, 123 in Web of Science Core Collection, 115 in EBSCO DOSS, and 222 in Google Scholar. The database search was supplemented by manual reference mining of relevant articles. In accordance with the SANRA recommendations, the selection of sources was based on their relevance to the main themes of the review, with a focus on recent chemical/technological innovations, clinical applications, and future trends. No formal inclusion or exclusion criteria or standardized quality assessment tools were used, as the aim was to synthesize and summarize key concepts and recent advances rather than conduct a systematic evaluation. With the exception of [4], 98 publications were included to support the narrative discussion.

## 3. Results and Discussion

### 3.1. “Gold Standard” in Enamel Bonding

**Figure 4:**
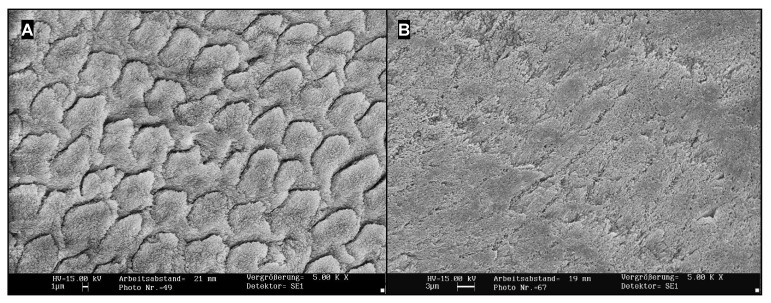
Scanning electron microscopy (SEM) micrographs showing (**A**) the enamel prisms after etching with 36% phosphoric acid (pH ≈ 0.8) and (**B**) a less pronounced etching pattern after self-etching of the prepared enamel (×5000 magnification per SEM micrograph).

Traditionally, enamel bonding is achieved by etching with 30–40% phosphoric acid, which dissolves hydroxyapatite and increases the enamel surface area and wettability [5]. Even ordinary hydrophobic bonding agents can infiltrate the etched and dried enamel by capillary attraction. Optimal micro-mechanical interlocking between polymerized resin and etched enamel via resin tag formation ensures a durable bond strength to enamel [5]. This classic phosphoric acid ER mode is still considered the benchmark for enamel bonding today and protects the more vulnerable adhesion to dentin against degradation phenomena [6,7]. Enamel has displayed higher bond strength when adhesives were applied in ER instead of SE mode due to a well-defined etching pattern and better micro-mechanical retention in enamel (Figure 4) [8,9].

Several authors highlighted the need for selective enamel etching with phosphoric acid to compensate for the insufficient etching and chemical bonding potential of acidic monomers on enamel [9,10,11]. The chemical (ionic) interaction between acidic monomers (i.e., phosphate, carboxylate, or phosphonate functional groups) and the hydroxyapatite in enamel is not as effective as in dentin, probably due to the different crystallinity in structure and/or size [6,12]. However, the streamlined workflow of universal adhesives may be questionable in the case of a separate etching and rinsing step (cf. Figure 3). In addition, conflicting clinical results (over a maximum of five years) exist on whether the bonding mode of universal adhesives (ER or SE) has a significant effect on Class V composite restorations in non-carious cervical lesions (NCCLs) [13,14,15,16,17]. To improve the enamel bond strength of adhesives, beveling of enamel margins has also been discussed in the literature [10,18,19].

### 3.2. Reconsidering Enamel Beveling in Posterior Cavities

Anterior and Class V cavities in visible areas are typically prepared with a bevel at the enamel margins to optimize the esthetic appearance through a gradual color transition between the composite restoration and the tooth (“chameleon effect”) [10]. It was also recommended to bevel the enamel margins in posterior cavities. The rationale is that beveling perpendicular to the enamel prisms not only increases the retentive surface of the cavity but also improves the effectiveness of phosphoric acid and acidic monomers in creating a micro-retentive etching pattern [20,21,22]. However, the clinical benefits of enamel beveling over non-beveled Class I and II cavities remain largely unproven in the literature [19,23]. The only two clinical studies (randomized controlled trials (RCTs)) on this topic reported that posterior composite restorations placed with or without enamel beveling performed equally well [24,25], with the exception of marginal discoloration, where one study demonstrated significantly better results in the beveled group after one year [25]. In addition, in vitro investigations suggested that proximal beveling of enamel margins has a negligible effect on marginal adaptation and microleakage of extended Class II composite restorations compared to box-shaped cavities, regardless of the bonding mode used (ER or SE) [26,27]. Apparently, the bonding potential of well-established adhesives combined with the low polymerization shrinkage of modern hybrid composites and/or incremental filling techniques has reduced or even eliminated any positive effect of enamel beveling [26,27,28]. Based on the limited clinical and in vitro data, the potential benefits may not outweigh the risks associated with beveling posterior cavities, such as proximal damage of adjacent teeth, application of excess composite, or marginal chipping due to thin composite extensions. Although no definitive clinical recommendation can be made, clinicians should carefully weigh these potential risks against the lack of strong clinical evidence supporting posterior beveling. Especially at the cervical margins of extended Class II cavities, priority should be given to enamel preservation over beveling to prevent loss of the already thinned enamel [26].

### 3.3. Gold Standard in Dentin Bonding

The bond strength to enamel is reportedly more durable than to dentin, since the host-derived hydrolysis and enzymatic degradation of hybrid layer components (i.e., polymerized resin and dentin collagen) cannot be prevented in the long term, regardless of which adhesive is currently used [7,29,30]. Adhesion to dentin has traditionally been achieved through a multi-step procedure involving etching, priming, and subsequent bonding prior to polymerization. Due to its hydrophilic nature, incompletely (SE) or fully (ER) demineralized dentin is difficult to infiltrate with hydrophobic monomers, requiring a hydrophilic primer to improve surface wetting and resin penetration into dentin collagen [6,29]. The principal hydrophilic monomer in adhesives is 2-hydroxyethyl methacrylate (HEMA), which acts as reactive diluent and promotes the diffusion of other monomers [30,31]. Single-bottle adhesives have simplified the classical concept by enabling faster bonding procedures with fewer steps, thus minimizing the time during which blood and saliva contamination could compromise the adhesive restoration. On the other hand, adopting simplified adhesives uncritically risks compromising the long-term success of restorations. Multi-bottle adhesives with primer and bonding resin in separate bottles, namely the three-step ER adhesive OptiBond FL (Kerr, Orange, CA, USA) and the two-step SE adhesive Clearfil SE Bond (Kuraray Noritake, Tokyo, Japan), are still considered the gold standard for dentin bonding. This belief was based on their clinical track record with low annual failure rates of Class V composite restorations in NCCLs after up to 14 years [6,32,33,34] as well as on in-vitro studies that showed superior performance compared to simplified adhesives in terms of dentin bond strength [28,35] and marginal quality of composite restorations [22,36]. The superiority has been corroborated by clinical results of RCTs and uncontrolled clinical trials, which showed higher retention rates of Class V composite restorations in NCCLs bonded with gold-standard adhesives than for those bonded with simplified counterparts in ER or SE mode [37,38,39,40]. Possible reasons included the fact that primer and bonding resin are applied separately, which improves dentin hybridization, the presence of the acidic monomer 10-methacryloyloxydecyl dihydrogen phosphate (10-MDP/MDP) in Clearfil SE Bond, or the high filler content of OptiBond FL (approximately 48% barium glass), which potentially enhances its mechanical properties [6,22,29,33,41].

### 3.4. Simplified Adhesive Protocols

**Figure 5:**
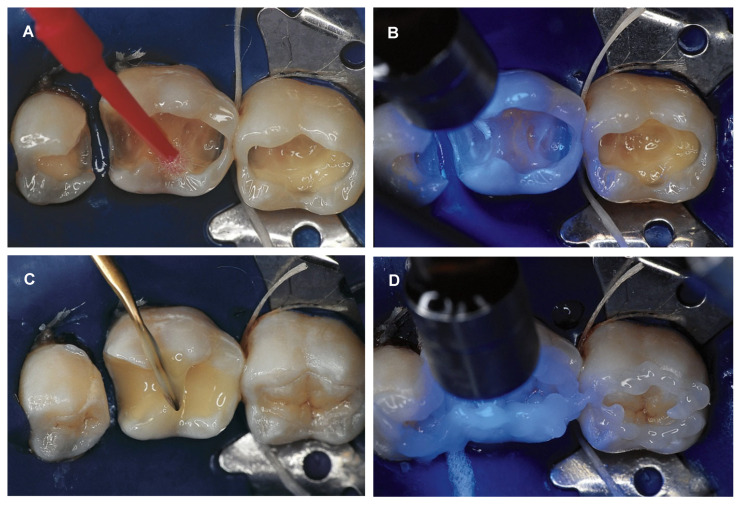
Bonding procedure when precuring a single-bottle adhesive with low layer thickness: (**A**) Application of the adhesive layer. (**B**) Separate light curing of the adhesive. (**C**) Application of a dual-curing luting composite. (**D**) Light curing after the insertion of glass–ceramic inlays.

Due to their intrinsic hydrophilicity, chemical mixtures of primer and bonding resin in a single bottle are expected to have a thinner and more hydrophilic adhesive layer, making it susceptible to suboptimal dentin hybridization, nanoleakage, and hydrolytic degradation of the hybrid layer [6,12,29]. However, there is no consensus in the literature regarding the ideal layer thickness. While in vitro data showed that thicker layers (by applying an additional layer or a filled adhesive) can form a stress-absorbing adhesive interface between the hybrid layer and the composite or improve dentin bond strength [22,36,42], thinner layers can be equally suitable for bonding if applied evenly to the dentin [43,44]. Clinical outcomes also remain inconclusive. The only three RCTs found on this topic reached different conclusions. Applying an additional layer either improved the short-term retention of Class V composite restorations in NCCLs after 18 months [45] or had no significant effect on their clinical performance after 18 months [46] and in the longer term (five years) [47], respectively. Thin layers can minimize the risk of adhesive pooling and radiographic misdiagnosis of secondary caries, or – if pre-cured - prevent possible misfits of indirect restorations. In vitro data suggested that dentin bonding under relatively thick ceramic restorations can be optimized by precuring the adhesives and using dual-curing luting composites [48]. However, the low layer thickness can lead to suboptimal polymerization due to oxygen inhibition, emphasizing the need for initiators that ensure a high conversion rate of the adhesive (Figure 5).

Other reported reasons for the limited bond durability of simplified adhesives in vitro include potential incompatibility with dual- or self-cure composites, increased water sorption and solubility of HEMA-rich adhesives, and monomer-solvent phase separation in formulations with low or no HEMA content [12,29,30,48,49]. Furthermore, mixing all the ingredients in a single bottle has led to issues with shelf stability. Some manufacturers therefore require that liquids from separate components or bottles be mixed before use in one step, while others have addressed this problem by using monomers that are more hydrolytically resistant such as acrylamides [30,50,51]. Universal adhesives have been associated with similar shortcomings, as they are commonly based on the single-bottle concept [6,12,30,52]. However, in vitro studies indicated that universal adhesives containing the acidic monomer MDP generally achieve higher and more durable dentin bond strength than single-bottle adhesives lacking this monomer [53,54,55]. Furthermore, the addition of MDP has been proposed to restore bond strength to dentin following saliva contamination [56], although other factors such as the type of solvent or the HEMA content may also play a role in how the bond strength of adhesives is affected by saliva [57,58]. Alternative or complementary acidic monomers to MDP used in single-bottle formulations include glycero-phosphate dimethacrylate (GPDM), 4-methacryloxyethyl trimellitic anhydride/4-methacryloxyethyl trimellitic acid (4-META/4-MET), and dipentaerythritol penta-acrylate phosphate (PENTA).

### 3.5. Chemical Bonding Using MDP

Due to its proven chemical bonding potential, MDP is not only the most common acidic monomer in adhesives but is also used in other applications such as ceramic-metal primers, surface cleaners for saliva decontamination, and self-adhesive composites [6,56,59]. Its dihydrogen phosphate group interacts chemically with the metal oxides on zirconium oxide and metal alloys, while the methacrylate group copolymerizes with resin monomers [60,61]. Tooth interaction of this monomer is based on the relatively strong etching effect for micro-mechanical interlocking and chemical bonding between the phosphate group and the calcium ions of the incompletely dissolved hydroxyapatite according to the so-called adhesion-decalcification concept [6,62]. The higher bond durability of MDP-based adhesives has been partly attributed to the deposition of hydrolytically stable crystals, which are arranged in calcium salt nanolayers, reinforcing the hybrid layer [53,59,63]. However, the clinical relevance of MDP nanolayering per se remains difficult to demonstrate. For example, the mode of action of MDP on phosphoric acid-etched dentin is not yet fully understood. While there is consensus that MDP contributes to the preservation of the hybrid layer by inhibiting degenerative enzymes such as matrix metalloproteinases MMP-2/9 and by hydrogen bonding with collagen molecules [64], it remains controversial whether MDP nanolayering can truly occur in hydroxyapatite-free ER hybrid layers [65]. In vitro studies indicated that the effectiveness of acidic monomers including MDP in caries-affected dentin may be compromised due to the lower mineral content and disorganized crystalline structure compared to sound dentin [62,66]. On the other hand, MDP-based universal adhesives performed better in ER mode than their MDP-free counterparts [55], leading researchers to conclude that a specific interaction must be occurring [67,68]. Furthermore, the effectiveness of MDP can be compromised by its concentration, impurities, and potential influence of other components in the adhesive formulation [69]. For example, the common ingredient HEMA is suspected of competing with MDP for calcium affinity, thereby impairing the chemical bonding and MDP nanolayering [51,70]. Moreover, both phosphate ester groups of MDP are susceptible to hydrolytic degradation, which may compromise the bond durability despite the initially favorable chemical bonding [51,68]. Recent developments in adhesive technology are aimed at overcoming these limitations. To mitigate the negative effects of HEMA, its content in acrylamide-based adhesives has been reduced or completely replaced. While HEMA-free adhesives are particularly prone to phase separation, acrylamide-based formulations (specifically those incorporating bis-acrylamides) have been able to solve this problem by acting as a co-solvent for other monomers that do not adequately mix with water, being commercially termed as “active guard technology” [50]. Furthermore, amide groups are recognized for being more hydrolytically stable and resistant to degradation than the ester groups of traditional methacrylates such as HEMA [51,71]. To address the low hydrolytic stability of MDP, researchers investigated alternatives for chemical bonding with higher hydrolytic resistance, such as novel fluoro-carbon phosphate (6-methacryloxy-2,2,3,3,4,4,4,5,5-octafluorohexyl dihydrogen phosphate (MF8P)) or acrylate- and siloxane-modified polyurethane (SPU) monomers [51,68,72]. However, to date, no commercially available adhesive formulation is exclusively hydrolytically stable, which makes it difficult to assess the clinical potential of such resin matrices.

### 3.6. Reconsidering Gold Standard Adhesives

While it is plausible that the merging of primer and bonding resin in a single solution creates inherent compromises, clinical evidence from RCTs in NCCLs showed that the performance of adhesives depends on a product level rather than the bonding mode used (ER or SE) or the number of bottles and steps [19,73,74,75,76,77]. The differences in performance could be explained by different chemical formulations and mechanical properties of the materials (e.g., filler content, specific monomers, or type of solvent), with some of them performing better than others in each adhesive class [76,77]. Apart from product dependency, non-material parameters such as clinician- and patient-related factors were also important [33]. In fact, the longest follow-up of adhesives in NCCLs published to date reported that a simplified adhesive strategy does not affect the clinical performance of Class V composite restorations over the period of 14 years [34]. Emerging evidence indicates that there may be no significant difference in the clinical success of gold standard and other adhesives (mostly single-bottle formulations) [75,76,78], which seems to refute the claim that simplified adhesive protocols attractive to clinicians necessarily lead to reduced performance. However, the body of evidence was considered rather low in these meta-analyses due to the small number of eligible RCTs with a follow-up of four years or more [75,76,78], which may not have been sufficient to distinguish clinically between the adhesives. Moreover, the RCTs included in the meta-analyses were limited to the treatment of NCCLs. Although it is generally accepted that non-retentive NCCLs of Eccles Class II or III type are more ideal for evaluating adhesives than prepared and/or excavated cavities that mask differences in retention, Class V composite restorations are subject to different requirements than load-bearing restorations [7]. Thus, clinical performance may not be equally transferable, particularly regarding postoperative hypersensitivity, marginal adaptation and discoloration. However, clinical trials comparing gold standard and simplified adhesives in cavities other than NCCLs are scarce in the literature and lack sufficient follow-up periods (Table 1). While current evidence shows comparable performance of the adhesives over one to three years, longer-term clinical studies are needed, especially for load-bearing Class II restorations, which represent a large proportion of restorative cavity configurations in clinical practice. Furthermore, practice-based clinical studies may be necessary to compare the performance in “true life” situations under less ideal clinical conditions than those found in controlled university settings.

### 3.7. Progress in Self-Adhesive Composites

**Figure 6:**
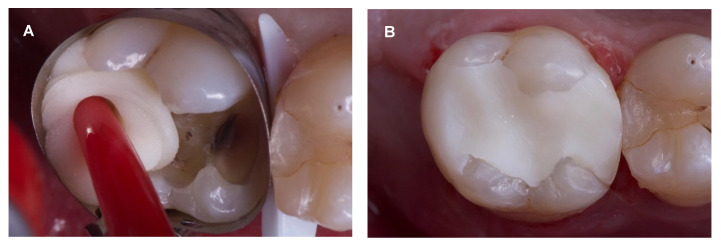
Streamlined restorative procedure using a self-adhesive bulk-fill composite: (**A**) Direct application into a Class II cavity. (**B**) Dual-curing of the restoration (Courtesy of Dr. L. Póti, Debrecen, Hungary).

Self-adhesive composites represent the next logical step toward simplifying the restorative procedure and are particularly useful in difficult-to-access cavities or for patients with special health-care needs who cannot tolerate prolonged treatments. Since these materials bond directly to enamel and dentin without the need for a separate adhesive, their resin matrices are modified through the incorporation of acidic monomers [79,80,81]. Self-adhesive flowable composites use monomers for bonding that are commonly found in adhesives. While GPDM and 4-META were key components in early products, more recent developments have focused on the use of MDP to enhance chemical bonding (via its superior calcium salt stability). In addition, all formulations contain HEMA or similar hydrophilic diluents [81], which make them susceptible to water sorption and solubility [79]. The water uptake could trigger hydrolytic cleavage of the polymer matrix and acidic monomers such as GPDM, MDP or 4-META, and/or cause plasticization of the resin. Over time, this degradation could manifest clinically as color instability [81]. Balancing the acidity required for self-etching with a sufficiently high filler content for mechanical strength has also led to some limitations. Following short-term clinical reports on the lack of retention when used in NCLLs or for fissure sealing [82,83], clinicians have been advised to apply self-adhesive flowable composites with rubbing motion to the cavity and/or to etch the enamel margins with phosphoric acid to improve bond strength [11,81]. However, their clinical success is largely confined to non-load-bearing areas, such as small Class I restorations, rendering these flowable formulations suitable only for niche applications due to their mechanical constraints [80,81]. Research is now focusing on self-adhesive composites that have a higher mechanical capacity than self-adhesive flowable composites and can be indicated for load-bearing restorations in the posterior region. These novel materials are mainly developed based on a combination of new acidic monomer technologies and bulk-fill composites through polymerizable acid polymers [84,85]. They utilize dual-curing mechanisms by combining light-curing at the surface with self-curing in deeper cavity areas, which enables bulk placement in a single step. While the slower self-curing can promote self-etching and reduce polymerization shrinkage stress, uncured acidic monomers may impair redox curing. Alternative initiator systems (e.g., persulfate with reducing agents) have been developed in conjunction with the photoinitiator champhorquinone to ensure complete polymerization of the bulk-filling (Figure 6) [84,85].

Nevertheless, conclusions regarding the clinical performance of self-adhesive bulk-fill composites must be interpreted with caution, as the underlying evidence is heavily weighted toward short-term observations. Clinical data spanning a period of six months to three years indicated that these self-adhesive composites achieve acceptable outcomes in Class I, II, and V restorations and perform comparably to composites bonded with separate adhesives. However, they showed less satisfactory esthetic properties regarding color match and translucency. A critical factor is the lack of strong clinical evidence for load-bearing restorations, as many clinical failures of restorative materials manifest late. Currently, there is only a single study reporting follow-up periods of up to five years in Class II cavities. Consequently, definitive conclusions regarding their clinical durability, marginal adaptation, and resistance to fracture or wear under occlusal loading in the posterior region can only be drawn once longer observation periods are published. At present, however, self-adhesive bulk-fill composites are not available on the dental market, which limits their availability for clinical use and further research (Table 2).

**Table 1:** Clinical trials comparing simplified adhesives with the gold standard in cavities other than Class V non-carious cervical lesions.

Study ID	Study Type	Sample Size	Cavity	Simplified Adhesive	Gold Standard Control	Evaluation Criteria	Outcome	Recall Period
Vinagre et al., 2020 [86]	RCT parallel	N_P_ = 54 N_R_ = 159	Class I	Prime & Bond NT (ER) Xeno III (SE) Xeno V (SE)	Clearfil SE Bond OptiBond FL	FDI	No significant difference between the adhesives, except better marginal adaptation for ER.	1 year
Delbons et al., 2015 [87]	RCT split mouth	N_P_ = 45 N_R_ = 144	Class I, II	OptiBond Solo Plus (ER) OptiBond XTR (SE) OptiBond All-in-One (SE)	OptiBond FL	Modified USPHS	No significant difference between the adhesives.	1.5 years
Özden & Karadas 2025 [88]	RCT parallel	N_P_ = 124 N_R_ = 252	Class II	G-Premio Bond (SE) Single Bond Universal (SE)	Clearfil SE Bond 2 *	FDI	No significant difference between the adhesives.	1.5 years
Ermis et al., 2009 [89]	RCT split mouth	N_P_ = 33 N_R_ = 87	Class II	Adper Single Bond (ER)	Clearfil SE Bond	Modified USPHS	No significant difference between the adhesives.	2 years
Donmez et al., 2016 [90]	RCT split mouth	N_P_ = 32 N_R_ = 128	Class II (primary teeth)	XP Bond (ER) AdheSE (SE) G-Bond (SE)	OptiBond FL	FDI	No significant difference between the adhesives, except better marginal adaptation for ER.	3 years
Comba et al., 2023 [91]	Retrospective clinical study	N_P_ = 90 N_R_ = 168	Class III, IV	Clearfil Universal Bond Quick (ER)	OptiBond FL	Modified USPHS	No significant difference between the adhesives.	~3 years

ER: Etch-and-rinse; FDI: World Dental Federation; N_P_: Number of patients; N_R_: Number of restorations; RCT: Randomized controlled trial; SE: Self-etch; USPHS: United States Public Health Service; (*) Successor of Clearfil SE Bond.

**Table 2:** Clinical trials evaluating self-adhesive bulk-fill composites.

Study ID	Study Type	Sample Size	Cavity	Self-Adhesive Bulk-Fill Composite	Composite/Adhesive Control	Evaluation Criteria	Outcome	Recall Period
Maghaireh et al., 2023 [92]	RCT split mouth	N_P_ = 83 N_R_ = 166	Class I, II	Surefil One *	Filtek Bulk Fill/ Adper Single Bond 2 (ER)	Modified USPHS & VAS	100% survival. No significant difference between the composites.	0.5 year
Rabie et al., 2025 [93]	RCT split mouth	N_P_ = 40 N_R_ = 80	Class I (primary teeth)	Surefil One	Grandio/Single-bottle adhesive by Bisco ** (SE)	FDI	No significant difference between the composites, except better esthetic properties of the control.	0.5 year
Ellithy et al., 2024 [94]	RCT split mouth	N_P_ = 32 N_R_ = 64	Class II	Surefil One	Filtek One Bulk Fill/ Scotchbond Universal (SE)	FDI	100% survival. No significant difference between the composites, except better esthetic properties of the control.	1 year
El-Shazly et al., 2025 [95]	RCT split mouth	N_P_ = 15 N_R_ = 54	Class V	Surefil One	Neo Spectra ST/ Prime & Bond Universal (SEE)	Modified USPHS & VAS	100% retention. No significant difference between the composites, except better esthetic properties of the control.	1 year
Sabry et al., 2024 [96]	RCT parallel	N_P_ = 40 N_R_ = 40	Class II	Surefil One	Tetric N-Ceram Bulk Fill/ Tetric N-Bond (SEE)	Modified USPHS	95% retention. No significant difference between the composites, except better esthetic properties of the control.	1.5 years
Albelasy et al., 2024 [97]	RCT split mouth	N_P_ = 32 N_R_ = 96	Class I, II	Surefil One	Powerfil/Adhese Universal (SEE) Cention N/Adhese Universal (SEE)	FDI	100% survival. No significant difference between the composites, except poorer marginal adaptation of Cention N restorations.	2 years
Rathke et al., 2025 *** [98]	PBRN	N_P_ = 41 N_R_ = 60	Class I, II, V	Surefil One	–	Modified USPHS	Three restorations failed. The others were clinically acceptable despite some color changes.	3 years
Schenke et al., 2025 *** [99]	RCT split mouth	N_P_ = 30 N_R_ = 60	Class II	SABF *	Filtek One Bulk Fill/ Scotchbond Universal (SE)	FDI	98.9% survival. No significant difference between the composites, except better esthetic properties of the control.	5 years

ER: Etch-and-rinse; FDI: World Dental Federation; N_P_: Number of patients; N_R_: Number of restorations; PBRN: Practice-based research network; RCT: Randomized controlled trial; SE: Self-etch; SEE: Selective enamel etching; USPHS: United States Public Health Service; VAS: Visual analogue scale; – Not applicable; (*) Currently not commercially available; (**) Adhesive brand not specified; (***) Study with the longest follow-up from the same clinical trial.

## 4. Future Direction and Concluding Remarks

Significant advances in the simplification and versatility of single-bottle adhesives have challenged the status of “gold standard” adhesives. Simplified adhesives can offer clinical advantages, for example, in scenarios with compromised isolation or less accessible cavities where procedural simplicity is paramount. Further developments are moving toward self-adhesive composites that combine self-adhesive properties with load-bearing capacity. Although simplified adhesives and self-adhesive composites have shown encouraging results, their long-term reliability in load-bearing restorations remains uncertain due to a lack of extended survival data. Drawing overall conclusions is further complicated by the limitations of existing meta-analyses on this topic, which are largely based on short-term RCTs in NCCLs and whose generalizability to load-bearing composite restorations (particularly in Class II cavities) is limited. Future clinical studies should also focus on practice-based research that evaluates whether these simplified workflows can reliably replace or even surpass complex restorative procedures in adhesive dentistry under “real-world” conditions and when performed by different dental practitioners. Beyond clinical performance, economic evaluations should be included to assess their cost-effectiveness and cost–benefit ratio. Looking to the future, the focus is on overcoming the degradation of the dentin hybrid layer. Advances include the integration of antimicrobial monomers to prevent secondary caries, the incorporation of remineralizing agents such as calcium phosphate particles to stabilize collagen fibrils, and the refinement of hydrophobic polymer matrices to enhance hydrolytic stability. The ultimate goal would be a modular “all-in-one” composite with bioactive properties that possesses both adhesive and self-adhesive properties and functions interchangeably as a restorative material or a luting cement [51]. This modularity would further streamline the restorative workflow and offer a versatile single-material solution that ensures high durability across various clinical indications.

## Data Availability

No new data were created or analyzed in this study.
